# MicroRNA Expression in Endometrial Cancer: Current Knowledge and Therapeutic Implications

**DOI:** 10.3390/medicina60030486

**Published:** 2024-03-15

**Authors:** Irene Iavarone, Rossella Molitierno, Pietro Fumiento, Maria Giovanna Vastarella, Stefania Napolitano, Maria Teresa Vietri, Pasquale De Franciscis, Carlo Ronsini

**Affiliations:** 1 Department of Woman, Child and General and Specialized Surgery, University of Campania “Luigi Vanvitelli”, 80138 Naples, Italy; ireneiavarone2@gmail.com (I.I.); molitiernorossella@gmail.com (R.M.); pietro.fumiento@virgilio.it (P.F.); mariagiovanna.vastarella@studenti.unicampania.it (M.G.V.); pasquale.defranciscis@unicampania.it (P.D.F.); 2 Division of Medical Oncology, Department of Precision Medicine, University of Campania “Luigi Vanvitelli”, 80138 Naples, Italy; stefania.napolitano@unicampania.it; 3 Department of Precision Medicine, University of Campania “Luigi Vanvitelli”, 80138 Naples, Italy; mariateresa.vietri@unicampania.it

**Keywords:** endometrial neoplasm, tumor microenvironment, microRNA, exosomes, cell-derived microparticle

## Abstract

*Background and Objectives*: An extracellular vesicle is part of a class of submicron particles derived from cells, mediating cellular crosstalk through microRNA (miRNA). MiRNA is a group of RNA molecules, each of which consists of 15–22 nucleotides and post-transcriptionally modulates gene expression. The complementary mRNAs—onto which the miRNAs hybridize—are involved in processes such as implantation, tumor suppression, proliferation, angiogenesis, and metastasis that define the entire tumor microenvironment. The endometrial biopsy is a standard technique used to recognize cellular atypia, but other non-invasive markers may reduce patient discomfort during the use of invasive methods. The present study aims to examine the distribution and the regulation of the differentially expressed miRNAs (DEMs) and EV-derived substances in women with endometrial cancer. *Materials and Methods*: We systematically searched the PubMed, EMBASE, Scopus, Cochrane Library, and ScienceDirect databases in April 2023, adopted the string “Endometrial Neoplasms AND Exosomes”, and followed the recommendations in the Preferred Reporting Items for Systematic Reviews and Meta-Analyses (PRISMA) statement. We selected all the studies that included patients with endometrial cancer and that described the regulation of miRNA molecules in that context. The differences in molecule expression between patients and controls were evaluated as significant when the proteins had a fold change of ±1.5. *Results*: Seventeen records fulfilled the inclusion criteria: a total of 371 patients and 273 controls were analyzed. The upregulated molecules that had the widest delta between endometrial cancer patients and controls—relative expression ≥ 1 > 3 log2(ratio)—were miR-20b-5p, miR-204-5p, miR-15a-5p, and miR-320a. In particular, miR-20b-5p and miR-204-5p were extracted from both serum and endometrial specimens, whereas miR-15a-5p was only isolated from plasma, and miR-320a was only extracted from the endometrial specimens. In parallel, the most downregulated miRNA in the endometrial cancer patients compared to the healthy subjects was miR-320a, which was found in the endometrial specimens. *Conclusions*: Although their epigenetic regulation remains unknown, these upregulated molecules derived from EVs are feasible markers for the early detection of endometrial cancer. The modulation of these miRNA molecules should be assessed during different treatments or if recurrence develops in response to a targeted treatment modality.

## 1. Introduction

Endometrial cancer (EC) is a malignancy of the inner epithelial lining of the uterus, with an increasing worldwide incidence and disease-associated mortality [1]. The global incidence of EC in 2020 was 417,336, and EC is the sixth most commonly occurring female cancer [2]. Most cases occur between 65 and 75 years of age [3]. Among perimenopausal and postmenopausal women, postmenopausal bleeding (PMB) accounts for approximately two-thirds of all gynecological visits and is a common symptom of EC [4]. For early-stage disease, the main treatment is surgery. Depending on the stage of the disease and other risk factors, adjuvant radiotherapy and/or chemotherapy can be added as an adjuvant treatment option to decrease the risk of relapse [5]. Surgical staging is used for prognostication and identification of women who might benefit from adjuvant treatment, according to the current stratification of patients according to risk [6]. Total hysterectomy with bilateral salpingo-oophorectomy (BSO) is the standard of care and can be performed using an open or minimally invasive approach [7,8,9]. Moreover, lymph node status must be investigated to address adjuvant treatment [10]. EC management remains challenging, and a deeper understanding of the genetic diversity as well as the drivers of the various pathogenic states of this disease has led to the development of divergent management approaches in an effort to improve therapeutic precision in this complex malignancy [11]. Recently, greater emphasis has been given to the molecular profile of the disease, but it still neglects to totally explain the differences in the evolution of EC subtypes [12]. A substantial difference in EC management and treatment may be caused by extracellular vesicles (EVs), which are a class of submicron particles derived from cells that mediate cellular crosstalk through microRNA. MicroRNA (miRNA) is a small non-coding RNA that binds to target messenger ribonucleic acid (mRNA) to inhibit post-transcriptional gene expression and plays an essential role in regulating gene expression, the cell cycle, the timing of biological development, etc. [13]. Complementary mRNA—onto which miRNAs hybridize—are involved in the implantation, tumor suppression, apoptosis, proliferation, angiogenesis, and metastasis that define the tumor microenvironment [14]. EVs have a role as carriers of molecular pathways. In that context, miRNAs are the most investigated substances in EVs [13]. Their sources are blood samples (to be intended as serum and/or plasma), biopsy specimens, urine samples, and cellular compartments. Many studies have revealed the role of miRNAs in the biological processes of various cancers and other conditions: these molecules can help clinicians detect the existence of cancer as early as possible and screen out those with undiagnosed suspicious cases and healthy people [15,16]. On the other hand, it may reproduce the concrete risk for disease recurrence in patients, which is still a major oncological issue [17]. The wide variation in the recurrence patterns of endometrial carcinoma, to date, shows no gold standard in the timing and modalities of investigation. Although major international guidelines suggest checks every four months during the first two years of disease, the most suitable method to prevent recurrence is unknown. Moreover, this scenario is complicated by the possibility of the disease recurring locally or at a distant site because of metastasis. Deepening our knowledge of miRNA expression in endometrial carcinoma may facilitate the diagnosis of recurrences and the prediction of ways in which the disease may recur, thus enabling clinicians to choose the most appropriate follow-up method. Several studies have shown that specific miRNAs and EV-derived molecules can be used as high-precision biomarkers for EC detection [18]. In light of this, circulating molecules show great potential for use as cancer biomarkers because of their stability in peripheral serum or plasma. Because blood samples are easy to obtain and blood testing is cheap and convenient, circulating biomarkers can be used alone or in combination with other traditional screening methods for preliminary screening before invasive pathological and imaging examinations. In fact, even though endometrial biopsy is a standard option used to diagnose cellular atypia, non-invasive markers may be useful in minimizing patient discomfort compared to invasive techniques. The present study aims to examine the distribution and regulation of differentially expressed miRNAs (DEMs) and EV-derived substances in EC-affected women and the potential use of these molecules in the clinical management of EC.

## 2. Materials and Methods

The methods for this work were specified a priori on the basis of the recommendations in the Preferred Reporting Items for Systematic Reviews and Meta-Analyses (PRISMA) statement [19]. The present review is categorized on the PROSPERO site for the meta-analyses with the following protocol number: ID437914.

### 2.1. Search Method

**Figure 1:**
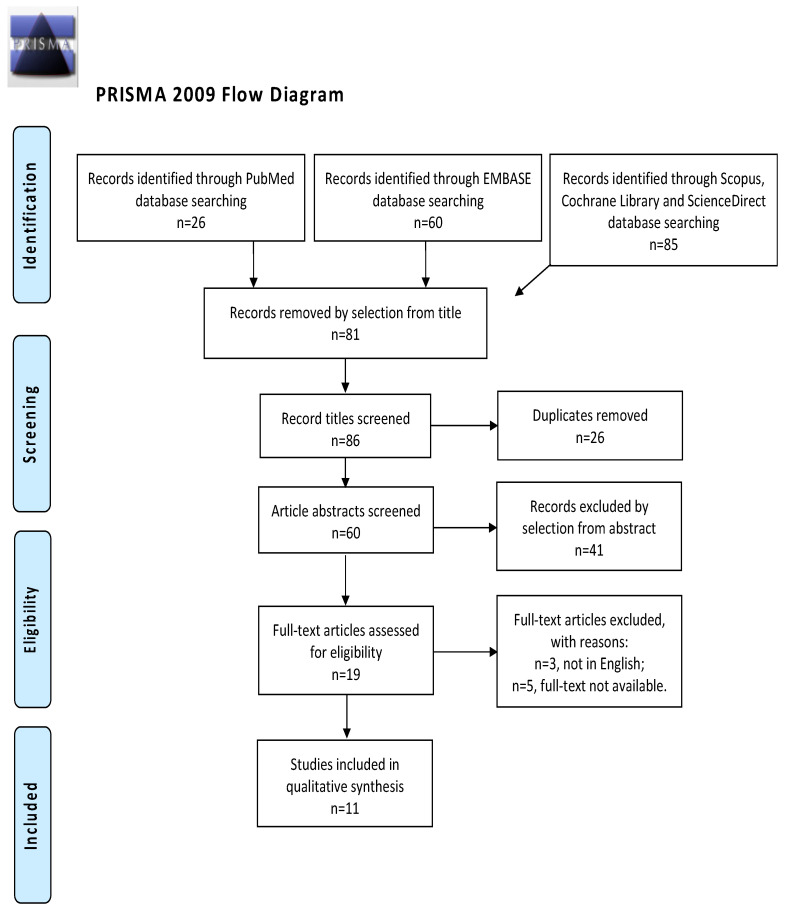
Preferred Reporting Items for Systematic Reviews and Meta-Analyses (PRISMA) flow diagram.

A systematic search was performed for articles in the PubMed database, Embase, Cochrane Library, ScienceDirect, and Scopus database in April 2023 using the string “Endometrial Neoplasms AND Exosomes”. There were no restrictions on the year of publication or the country of origin, and only articles published in English were taken into account (Figure 1).

### 2.2. Study Selection

The study selection process was carried out by I.I. and R.M. independently. In cases of discrepancies, C.R. established the inclusion or exclusion of the article in question. The inclusion criteria were the following: (1) studies that included patients with EC and described miRNA regulation in the context of that neoplasm; (2) studies reporting the outcome of interest, such as miRNA regulation in EC, and its clinical implication; and (3) all peer-reviewed articles that were originally published. We excluded preclinical trials, unoriginal studies, animal trials, articles in languages other than English, and abstract-only publications. When possible, authors of different studies that were only published as conference abstracts were contacted through e-mail and asked to show their final results. We mentioned all selected studies and the reasons for their exclusion in the Preferred Reporting Items for Systematic Reviews and Meta-Analyses (PRISMA) flowchart (Figure 1). We assessed all the studies concerning potential conflicts of interest.

### 2.3. The Extraction and Quantification of miRNAs

Liquid biopsy is a technique used to isolate microvesicles from serum using a minimally invasive procedure [18]. The isolation, amplification, and quantification of miRNAs were carried out using various methods.

#### 2.3.1. Extracellular Vesicles: Definition and Classification

EVs are small vesicles that can be released by cells in various contexts [20,21,22,23,24,25,26,27,28]. They are subdivided into exosomes, microvesicles, and apoptotic bodies. Exosomes (30 to 100 nm) form endosomes, microvesicles (100 to 1000 nm) come from the plasma membrane, and apoptotic bodies are 0.1 to 5 μm in diameter [29]. Exosomes derive from multivesicular bodies (MVBs), and the “endosomal sorting complex required for transport” protein complex can regulate the process of release [30]. Next, MVBs fuse with the cellular membrane, releasing the particles. Immunoelectron microscopy has been used to assess tetraspanins such as CD9, CD63, and CD81 as crucial elements in exosomes because they can be used as markers [31,32,33,34,35,36,37,38]. In parallel, apoptotic bodies show positivity for caspases 3 and 7 [39]. Recently, EVs have been classified on the basis of diameter as small (30–100 nm) or medium/large (100–200 nm) [40].

#### 2.3.2. Extracellular Vesicles: Methods of Identification and Analysis

EV-specific proteins (e.g., tetraspanins) are detected by immunoblotting to assess EVs in samples [29]. Transmission electron microscopy (TEM), scanning EM, and cryogenic TEM can be used to recognize EV characteristics [30,31,32,33,34,35], whereas atomic force microscopy is used to evaluate the stiffness and elasticity of EVs [36,37,38]. Size distribution and polydispersity in biologic samples are analyzed using dynamic light scattering [39]. Nanoparticle tracking analysis detects dimensions and concentration through Brownian motion and scattered light or emitted fluorescence [40]. Tunable resistive pulse sensing identifies modifications in electricity as each of the EVs passes through an adjustable nanopore [41,42]. Moreover, asymmetric field-flow fractionation separates EVs according to hydrodynamic size down to the nanometer level [43]. The most feasible method for analysis is flow cytometry (FC) [44,45,46,47]. Polychromatic FC employs tracers that stain intact EVs and assesses their immunophenotypic characterization through the use of antibodies [48,49,50,51,52,53].

## 3. Results

### 3.1. Study Characteristics

After the database search, 86 studies matched the search criteria. After deleting duplicates and records with no full-text and incorrect study designs (e.g., reviews), 19 were eligible. Of those, 11 matched the inclusion criteria and were included in the final systematic review schema. Those data are summarized in Figure 1. Seven articles were prospective case-control studies evaluating miRNA expression in patients and controls; two records were prospective cohort studies; and two articles were retrospective studies. The countries where the studies were conducted, publication year, study design, number of participants, substance instilled, and procedure characteristics are included in Table 1. The publication years ranged from 2015 to 2021. A total of 371 patients with EC and 273 controls were included. The FIGO stage of disease ranged from I to IV.

### 3.2. Outcomes

#### 3.2.1. Early Diagnosis

A total of 19 miRNAs were upregulated and 6 were downregulated in women with EC. Those results are included in Table 2 and Table 3. Five of the records did not contain the FIGO stage of disease, but the six remaining studies did: two involved patients with stage I disease, two involved patients with disease in stages II and III, and two included patients with disease in stages I–IV. Particularly, Li et al. enrolled 23 women with stage I disease and showed that miR-148b was downregulated in cancer-associated fibroblasts (CAFs) [55]. In parallel, Zhou L. et al. enrolled 25 patients with stage I disease and showed that miR-765 was downregulated in endometrial specimens [64]. The studies by Záveský et al. and Zhang et al. each contained 10 enrolled patients with stage II–III and showed that miR-106b and miR-320a were downregulated in urine samples and endometrial specimens, respectively [54,60]. In studies involving patients with disease in stages I–IV, ECs were isolated from both blood samples and endometrial specimens and contained the following upregulated miRNAs: miR-381-3p, miR-143-3p, miR-195-5p, miR-20b-5p, miR-204-5p, miR-423-3p, and miR-484 [57,61].

#### 3.2.2. Endometrial Cancer-Specific miRNAs

Among the DEMs, the miRNAs with the widest delta between patients and controls—relative expression ≥ 1 > 3 log2(ratio)—were miR-20b-5p, miR-204-5p, miR-15a-5p, and miR-320a [60,61,63]. In particular, miR-20b-5p and miR-204-5p were extracted from both serum and endometrial specimens, whereas miR-15a-5p was only isolated from plasma, and miR-320a was only extracted from the endometrial specimens [60,61,63]. The only downregulated miRNA was miR-320a in the Zhang et al. study, and it was extracted from endometrial specimens and had a 1.25 delta between patients and controls [62]. Those results are summarized in Table 2 and Table 3.

## 4. Discussion

Considering the functional aspects of miRNAs, these molecules participate in intercellular crosstalk in the healthy and neoplastic endometrium [14,15,65]. Evidence has highlighted the potential role of miRNAs as important markers in EC patients. Hypothetically, miRNAs may form in an intracellular environment, leading to the release of ECs from various cells [66,67,68]. Although it is very complicated to establish which specific miRNAs are involved in the genesis of EC, miRNA expression in women undergoing a hysteroscopy or blood test has been underlined because miRNA analysis in these women may identify conditions that are difficult to diagnose [69,70]. These miRNAs may represent the first building blocks in the evolution of cancer pathology. A deep understanding of the tumor microenvironment can help elucidate the actual personal risk of cancer progression to more compromised disease states and can help tailor treatments for these patients. For example, lymph node status is crucial for disease management in these patients. The ascertainment of lymph node status has been made easier through the use of the sentinel lymph node [71,72,73], but the lymph node positivity range in patients with early lesions is highly variable, and little is known about the actual mode of progression of cancer dissemination in lymph nodes [74,75]. MiRNAs might play a role in this. Another application of miRNAs is the elucidation of tumor aggressiveness. The identification of upregulated or downregulated miRNAs may help clinicians monitor the transition from pretumor lesions, such as atypical hyperplasia, to established tumors. Similarly, to date, clinical decisions are made on the basis of postoperative risk factors, making treatment personalization difficult, e.g., for patients who want to undergo fertility sparing treatment (FST). To date, the absence of myometrial infiltration and a low tumor grade are the only permissive conditions for FST, but they do not necessarily represent the actual risk for those women and are difficult to ascertain preoperatively because of the fallibility of current imaging methods and the possibility that the biopsy sample is not representative of the tumor as a whole [6]. Reassuring results were obtained even in patients with grade 2 tumors, which has already been demonstrated by our group. This may be related to an incomplete understanding the microscopic mechanisms of tumor invasiveness in which miRNAs may play a crucial role [75]. Because the data in the scientific literature are heterogeneous, it would be useful to pay attention to the DEMs with the widest delta in terms of relative expression between patients and controls to reduce false positives in the extraction process. Downregulated miR-320a had a 1.25 delta between patients and controls and was isolated in endometrial specimens [60]. Those data show how miRNA modulation is likely influenced by the site of expression. The real focus of miRNA applications should be on increasing the ability of clinicians to diagnose early-stage disease [76]. All the miRNAs and molecules summarized in Table 2 and Table 3 originate from tumor-derived exosomes via RNA sorting and delivery. Those mechanisms allow sampling from other compartments, like serum or plasma. Obviously, as a primary method, liquid biopsy mainly via the serum of patients is more feasible and cost-effective. Liquid biopsy could be part of screening programs in both fertile and postmenopausal women, even in the absence of symptoms [77,78]. However, that needs further investigation into miRNA modulation, particularly in early-stage EC. For all those reasons, the best candidates for early detection of EC should be upregulated miRNAs [61,63]. Unfortunately, those miRNAs can be overexpressed in disease stages I to IV, underlining a potential difficulty in early diagnosis [61,63,79]. In addition, miRNAs could be used during follow-ups for women undergoing different therapeutic regimens. In that context, fluctuations in miRNA expression could clarify patient responses to therapy, allowing clinicians to optimize treatment protocols [79,80]. Furthermore, miRNA expression can help clinicians predict the pattern of recurrence and personalize treatment for patients [81]. The present work examined all the existing literature on this topic. That can be considered both a strength and a weakness because there is great heterogeneity of results in the literature. Another strength is that this topic could spur further studies about the most specific miRNAs that can be used as biomarkers in the treatment of endometrial neoplasms in the clinic. Recently, great emphasis has been placed on the molecular profile of these tumors, highlighting how mismatch repair defects and microsatellite instability may have prognostic weight [82]. MiRNA identification may be a remote signature of the underlying genomic substrate, but no data about it are reported in the literature. Therefore, in our view, future studies should be aimed at a more careful sample stratification to profile the actual risk categories of patients.

Further strategies to develop methods of early EC diagnosis could involve the study of microbiota in different compartments, which is similar to studies that have been conducted in other contexts, like endometriosis [83]. For example, concerning the microbial composition of patients with gynecological disorders, conclusive results in the scientific literature detail an existing interplay between the immune system, the neuroendocrine system, and the gut, which all participate in this pathogenesis [84,85]. Changes or potential alterations in the gut microbiota that can cause dysbiosis can also modify the pathways regulating immunosurveillance [86]. Microbes in the endometrial mucosa are less frequently studied, and that includes those in the entire female reproductive tract (FRT), even though there is a lack of agreement regarding the microbial composition in the FRT; hence, there are not enough data supporting microbial composition as a feature of cancer genesis, as is the case with other gynecological diseases [86]. On the other hand, the immune response can include imbalances in the pathways of cellular epigenetic regulation and augmented levels of proinflammatory cytokines in the serum of patients. In addition, the replication and diffusion of neoplastic cells can parallel the endurance of the abovementioned contexts, resulting in chronic inflammation in the tumor microenvironment, which will lead to angiogenesis and cell adhesion over time [85]. That is facilitated by the suppression of the cell-mediated immune response and by the participation of the following molecules and interleukins (ILs): IL-6, IL-8, and vascular endothelial growth factor (VEGF). These molecules are able to establish both the primary constitution and the duration of the disease [86]. Moreover, the microbiota regulates proteolysis, for example, serotonin is derived from tryptophan, and dopamine, noradrenaline, and adrenaline are synthesized from tyrosine [87]. The decarboxylation of tryptophan aids in the synthesis of tyrosine. At the same time, the uptake of tryptophan can activate the acute phase reaction (APR), generating inflammation [88,89]. In dysbiosis, the microbial composition changes, especially in terms of microbial enzymatic activity [87,88,89], whereas a eubiotic microenvironment consists of active vitamin (such as cobalamin) biosynthesis and an intense catabolism of xenobiotics, which could be implicated in the inflammatory process [87]. The recognition of key microbiota would pave the way for new diagnostic and therapeutic strategies, including both prebiotics and probiotics, that can be implemented before considering surgical approaches. That would be crucial in helping patients avoid hysterectomy; fertility-sparing techniques can also be considered [90,91,92]. Lastly, more knowledge regarding miRNAs may help clinicians better understand the natural evolution of this disease. The risk of EC spreading via lymphatic, hematologic, or contiguous routes may influence treatment options in terms of molecular profile, adjuvant therapy, and personalizing surgery with procedures such as lymphadenectomy, omentectomy, or adnexectomy [81,92,93,94,95,96]. Moreover, we call attention to other novel molecular signatures, such as circular RNAs and proteins, that are different among EC patients and healthy control subjects [97,98]. In particular, the proteins apolipoprotein A-I (APOA1), hemoglobin subunit beta (HBB), carbonic anhydrase 1 (CA1), hemoglobin subunit delta (HBD), apolipoprotein(a) (LPA), serum amyloid A-4 protein (SAA4), platelet factor 4 variant (PF4V1), and apolipoprotein E (APOE) were upregulated in the serum of EC patients compared to controls [97]. In parallel, the circular RNAs hsa-circ-0109046 and hsa-circ-0002577 were upregulated in the serum of EC-affected women [98], whereas the circular RNA hsa-miR-200c-3p was slightly upregulated in urine [99]. The latter approach could be employed in future studies.

## 5. Conclusions

Many recent studies have demonstrated that intercellular crosstalk is crucially important in EC, even though data in the literature are heterogeneous. A panel of miRNAs could be feasible for the early detection and progression of disease in EC-affected patients using liquid and endometrial biopsies even though there is a lack of evidence showing that DEMs can be used as biomarkers to guide various treatment protocols. In conclusion, although pathways of epigenetic regulation are unclear, the evaluation of miRNA expression is a valuable and cost-effective option in the diagnosis of EC. Further evidence is needed to clarify miRNA regulation during the remission, relapse, and progression of EC to facilitate the planning of a targeted disease management strategy.

## Figures and Tables

**Table 1:** Characteristics of included studies.

Author, Year of Publication	Country	Enrollment Period	Study Type	FIGO Stage of Disease	No. of Patients	No. of Controls
Záveský, 2015 [54] ^	Czech Republic	N/A	Prospective multicenter case-control	II–III	10	13
Li, 2018 [55]	China	N/A	Prospective monocenter case-control	I	23	23
Roman-Canal, 2019 [56]	Spain	N/A	Prospective monocenter case-control	N/A	25	25
Jia, 2020 [57]	China	2017–2019	Prospective monocenter cohort	I–IV	62	0
Jing, 2020 [58]	China	2018–2019	Prospective monocenter case-control	N/A	5	5
Shi, 2020 [59]	China	N/A	Retrospective monocenter cohort study	N/A	79	12
Zhang, 2020 [60]	China	N/A	Retrospective multicenter case-control	II–III	10	10
Fan, 2021 [61]	China	2016–2017	Prospective multicenter case-control	I–IV	92	102
Gu, 2021 [62]	China	N/A	Prospective monocenter case-control	N/A	25	25
Zhou W.J., 2021 [63]	China	2018–2019	Prospective monocenter cohort	N/A	15	15
Zhou L., 2021 [64]	China	N/A	Prospective monocenter case-control	I	25	31

^: sub-analysis of entire cohort. FIGO: International Federation of Gynecology and Obstetrics; N/A: not available.

**Table 2:** Upregulated miRNA expression profile in patients with endometrial cancer.

Author, Year of Publication	MicroRNAs	Source	Regulation in Endometrial Cancer	*Delta* Patients vs. Controls
Roman-Canal, 2019 [56]	miR-383-5p miR-10b-5p miR-34c-3p miR-449b-5p miR-34c-5p miR-200b-3p miR-2110 miR-34b-3p	Peritoneal lavage	Up	0.96 0.92 0.89 0.93 0.92 0.92 0.90 0.90
Jia, 2020 [57]	miR-381-3p	Endometrial specimen	Up	N/A
Shi, 2020 [59]	miR-133a	Endometrial specimen	Up	0.40
Fan, 2021 [61]	miR-143-3p miR-195-5p miR-20b-5p miR-204-5p miR-423-3p miR-484	Serum and endometrial specimen	Up	0.75 0.95 1.12 1.03 0.68 0.79
Zhou W.J., 2021 [63]	miR-15a-5p miR-106b-5p mi-R107	Plasma	Up	1.02 0.50 0.25

N/A: not available.

**Table 3:** Downregulated miRNA expression profile in patients with endometrial cancer.

Author, Year of Publication	MicroRNAs	Source	Regulation in Endometrial Cancer	*Delta* Patients vs. Controls
Záveský, 2015 [54] ^	miR-106b	Urine	Down	N/A
Li, 2018 [55]	miR-148b	CAFs	Down	0.70
Jing, 2020 [58]	miR-499a-5p	Endometrial specimen	Down	0.80
Zhang, 2020 [60]	miR-320a	Endometrial specimen	Down	1.25
Gu, 2021 [62]	miR-139-5p	Endometrial specimen	Down	0.58
Zhou L., 2021 [64]	miR-765	Endometrial specimen	Down	0.75

^: sub-analysis of entire cohort. CAFs: cancer-associated fibroblasts; N/A: not available.

## Data Availability

Data supporting the findings of the study are available in the References section.
